# Breastfeeding and interdental spacing in primary dentition: a digital cross-sectional study

**DOI:** 10.3389/fdmed.2026.1716233

**Published:** 2026-03-30

**Authors:** Paula Boo-Gordillo, Maria Fernanda García-Morales, Laura Marqués-Martinez, Carla Borrell-García, Clara Guinot-Barona, Juan Ignacio Aura-Tormos, Esther García-Miralles

**Affiliations:** 1 Doctoral School, Faculty of Medicine and Health Sciences, Catholic University of Valencia, Valencia, Spain; 2 Department of Dentistry, Medicine and Health Sciences Faculty, Catholic University of Valencia, Valencia, Spain; 3 Department of Stomatology, Medicine and Dentistry Faculty, University of Valencia, Valencia, Spain

**Keywords:** breastfeeding, deciduous teeth, dental arch, diet, digital image, malocclusion, oral habits

## Abstract

**Background:**

Interdental spacing in the primary dentition is a key physiological feature that facilitates proper eruption and alignment of permanent teeth. While genetic factors play a central role, environmental influences such as early feeding practices may also affect jaw development and spacing, although quantitative evidence is limited.

**Objective:**

To analyze the relationship between early feeding practices—including breastfeeding, formula feeding, bottle and pacifier use, and timing of complementary feeding—and interdental spacing in the primary dentition.

**Methods:**

A descriptive cross-sectional study was conducted with 52 children aged 3–5 years. Digital intraoral scans were obtained using an iTero Element 2® scanner, and 18 interdental spaces per child were digitally measured using Orthocad® software. Parents completed structured questionnaires detailing their children's feeding history. Data were analyzed using descriptive statistics and comparative tests.

**Results:**

The largest mean interdental spaces were observed in the primate areas, specifically between the left lateral incisor and canine in the maxilla (0.756 mm) and between the left canine and first molar in the mandible (0.356 mm). No statistically significant associations were found between feeding variables and interdental distances (*p* > 0.05). However, a consistent trend toward larger interdental spaces was observed in children with prolonged breastfeeding compared to those with shorter or no breastfeeding, in line with proposed physiological mechanisms.

**Conclusion:**

Early feeding practices were not significantly associated with interdental spacing in the primary dentition. Nevertheless, the observed trends suggest that prolonged breastfeeding may positively influence interdental spacing, highlighting the need for larger and longitudinal studies to further investigate the role of environmental factors in dental arch development.

## Introduction

The presence of interdental spaces in the primary dentition is a fundamental physiological characteristic that facilitates the eruption and proper alignment of the permanent teeth (1). Adequate spacing within the primary dental arches plays a crucial role in establishing normal occlusion and contributes to favorable functional, aesthetic, and oral health outcomes (2). Insufficient spacing during early childhood is frequently associated with crowding and malposition of permanent teeth, highlighting the importance of factors that influence jaw and dental arch development.

Although genetic determinants largely govern craniofacial growth, environmental factors also contribute significantly to jaw and dental arch development (3). Among these, diet and early feeding practices are particularly relevant due to their influence on masticatory function and muscular activity during critical growth periods. Diets requiring greater chewing effort are thought to stimulate jaw development and support adequate dental arch formation, whereas softer, processed diets may reduce functional stimuli and negatively affect arch development (4–6).

The primary dentition exhibits characteristic spatial features, including anterior and posterior interdental spaces and primate spaces, which reflect normal physiological development (7, 8). These features are considered indicators of adequate arch dimensions and growth patterns. Feeding behaviors that promote functional activity, such as breastfeeding and appropriate timing of complementary feeding, may support favorable orofacial development, while the absence of such stimuli may contribute to altered growth patterns (9–17).

Despite growing interest in the role of feeding practices in occlusal development, there remains a lack of precise, quantitative evidence regarding their direct influence on interdental spacing in the primary dentition, particularly using digital measurement techniques (18, 19). Therefore, the objective of this cross-sectional study was to quantitatively analyze the relationship between early feeding practices—including breastfeeding, formula feeding, bottle and pacifier use, and timing of complementary feeding—and interdental spacing in the primary dentition using digital intraoral scans. We hypothesized that children with a history of prolonged breastfeeding and later introduction of complementary foods may exhibit greater interdental spacing than those with different feeding patterns. The present study aims to provide data that may clarify the subtle influence of early feeding practices on interdental arch development.

## Materials and methods

### Study design and ethical approval

A descriptive cross-sectional study was conducted between September 2022 and April 2023 at the University Dental Clinic of the Universidad Católica de Valencia San Vicente Mártir. The study protocol was reviewed and approved by the institutional Ethics Committee (UCV/2021-2022/185) and was conducted in accordance with the Declaration of Helsinki. Written informed consent was obtained from the parents or legal guardians of all participating children prior to inclusion in the study.

### Study population

The study sample consisted of 52 preschool children aged between 3 and 5 years who attended the University Dental Clinic for routine dental care. Participants were recruited consecutively during the study period.

### Inclusion criteria

Children were included if they presented complete primary dentition, no erupted first permanent molars, absence of dental anomalies, crowns, restorations, or severe caries, adequate cooperation for intraoral scanning, and completion of the parental questionnaire.

### Exclusion criteria

Children were excluded if they had physical or psychological limitations affecting cooperation, premature loss of primary teeth, fixed orthodontic appliances, or chronic systemic diseases that could influence growth or dental development.

### Questionnaire on feeding practices

Parents or legal guardians completed an anonymous structured questionnaire designed by the principal investigator to collect information on early feeding practices. The questionnaire was reviewed by three professors of dentistry and two medical professors to ensure content validity. It included closed-ended questions assessing breastfeeding (duration and exclusivity), formula feeding, mixed feeding, age at introduction of complementary feeding, weekly dietary patterns, and the use of bottles and pacifiers. Responses were recorded using categorical or ordinal scales and subsequently coded for statistical analysis. Although no formal psychometric validation was performed, a pilot test was conducted with five parents to assess clarity and comprehensibility of the items. The complete questionnaire is provided as Supplementary Material S1.

### Measurement of interdental spaces

**Figure 1:**
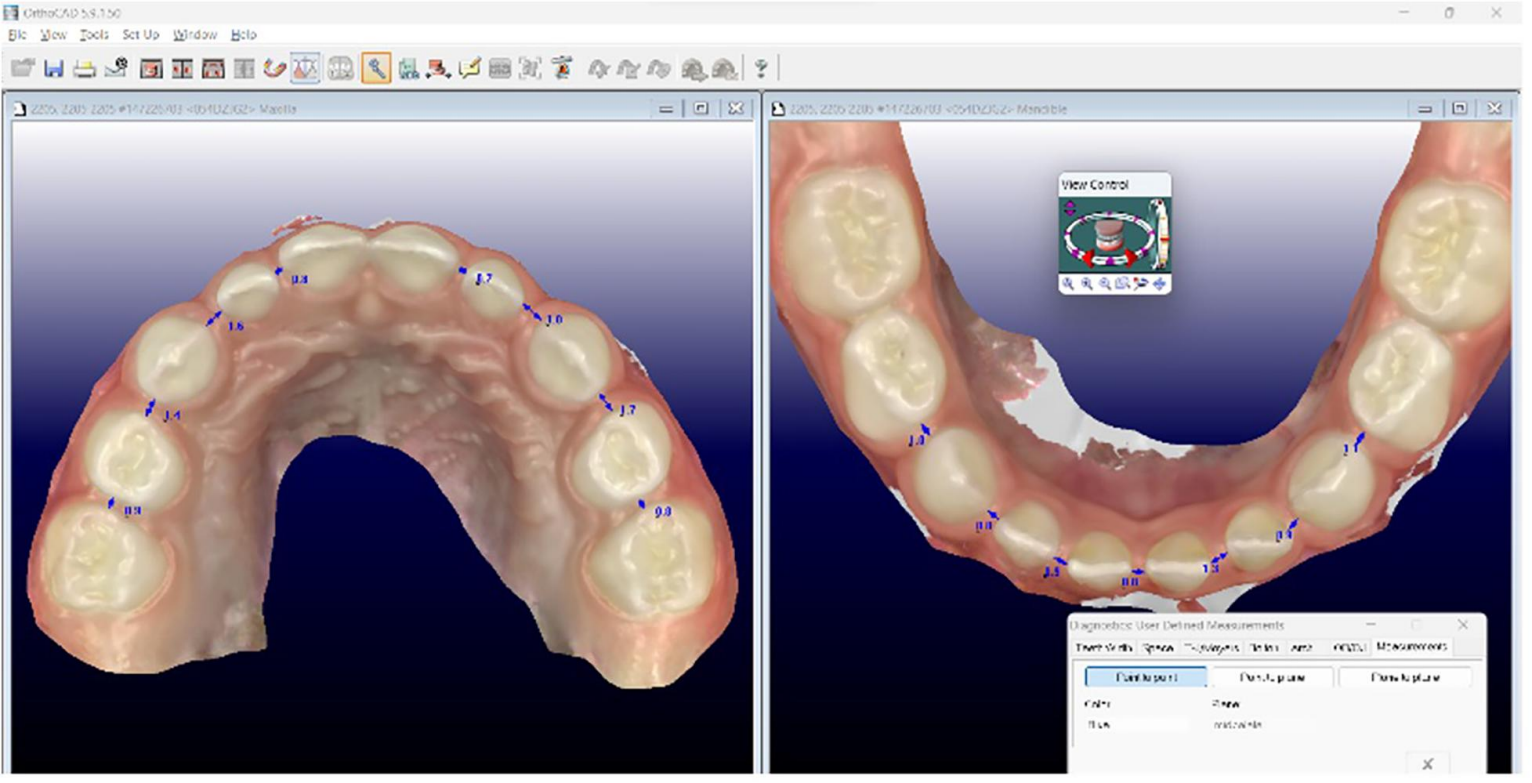
Measurement of interdental spaces using Orthocad®. Digital intraoral scans were obtained and interdental distances were measured at 18 reference points in the maxilla and mandible.

Digital intraoral scans of both maxillary and mandibular arches were obtained using an iTero Element 2® scanner (Align Technology, San Jose, CA, USA). All scans were performed by the same calibrated operator to minimize variability. Interdental distances were measured in millimeters using Orthocad® software. A total of 18 interdental spaces per child were assessed (nine per arch), including the following reference points: central–lateral incisors, lateral incisor–canine, canine–first molar, first–second molars, and the midline diastema. The software allowed precise visualization and identification of interdental diastemata (Figure 1).

### Outcome variables

The primary outcome variable was the mean interdental distance at each reference point in the maxillary and mandibular arches.

### Statistical analysis

Statistical analyses were performed using Python version 3.8.10 with pandas v1.3.3 and SciPy v1.7.1. Data distribution was assessed using the Shapiro–Wilk test, and homogeneity of variances was evaluated with Levene's test. Two-group comparisons (e.g., breastfed vs. non-breastfed children) were conducted using Student's *t*-test, Welch's *t*-test, or the Mann–Whitney *U* test, as appropriate. Comparisons among more than two groups (e.g., breastfeeding duration categories: <6, 6–12, >12 months) were performed using one-way ANOVA or the Kruskal–Wallis *H* test depending on data assumptions. Results are reported as means ± standard deviation for normally distributed variables or as medians and interquartile ranges for non-normally distributed data. Missing data were minimal (<5%) and handled using pairwise deletion. Statistical significance was set at *p* < 0.05. No *a priori* sample size calculation was performed, which is acknowledged as a limitation of the study.

## Results

### Interdental spaces and feeding practices

A total of 52 children aged 3–5 years (mean 51.8 ± 8.5 months; 55.8% boys) were included. Table 1 shows the mean interdental distances and standard deviations for all measured sites in the maxilla and mandible. In the maxilla, the largest mean interdental distance was observed between the left lateral incisor and canine (0.756 mm), while in the mandible it was between the left canine and first molar (0.356 mm). The greatest variability was found in the maxillary midline diastema (SD = 0.639 mm) and the mandibular canine–first molar space (SD = 0.398 mm).

**Table 1:** General means and standard deviations of interdental spaces.

No.	Arch	Interdental space	Mean (mm)	SD (mm)
1	Maxilla	Right first–second molar	0.072	0.217
2	Maxilla	Right canine–first molar	0.378	0.374
3	Maxilla	Right lateral incisor–canine	0.656	0.472
4	Maxilla	Right central–lateral incisor	0.310	0.270
5	Maxilla	Midline diastema	0.408	0.640
6	Maxilla	Left central–lateral incisor	0.288	0.253
7	Maxilla	Left lateral incisor–canine	0.756	0.519
8	Maxilla	Left canine–first molar	0.420	0.411
9	Maxilla	Left first–second molar	0.066	0.184
10	Mandible	Right first–second molar	0.034	0.101
11	Mandible	Right canine–first molar	0.346	0.399
12	Mandible	Right lateral incisor–canine	0.214	0.291
13	Mandible	Right central–lateral incisor	0.304	0.352
14	Mandible	Midline diastema	0.276	0.347
15	Mandible	Left central–lateral incisor	0.368	0.364
16	Mandible	Left lateral incisor–canine	0.272	0.294
17	Mandible	Left canine–first molar	0.356	0.389
18	Mandible	Left first–second molar	0.030	0.092

### Dietary and feeding habits

Dietary and feeding habits are summarized in Table 2. Breastfeeding was reported in 73.1% of children, with 48% continuing beyond 6 months. Formula feeding was reported in 67.3%, 44.3% received mixed feeding, 83% used a bottle, 54% used a pacifier, and 96.2% had complementary feeding introduced.

**Table 2:** Summary of dietary and feeding habit variables (*n* = 52).

Variable	*n*	%
Sex (male)	29	55.8
Sex (female)	23	44.2
Breastfeeding (yes)	38	73.1
Breastfeeding (no)	12	23.1
Formula feeding (yes)	35	67.3
Formula feeding (no)	17	32.7
Bottle use (yes)	43	82.7
Bottle use (no)	9	17.3
Dummy use (yes)	28	53.8
Dummy use (no)	24	46.2
Complementary feeding (yes)	50	96.2
Complementary feeding (no)	2	3.8

### Associations between feeding practices and interdental spacing

Mean interdental distances by feeding practice are shown in Table 3. Across all feeding variables, maxillary interdental spaces were consistently larger than mandibular spaces. Children breastfed for more than 6 months exhibited a tendency toward greater interdental spacing in both arches compared with those breastfed for shorter durations, although differences were not statistically significant (*p* > 0.05). Slight variations were observed for pacifier and bottle use, but no consistent patterns or statistically significant associations were detected. Formula feeding and complementary feeding were not associated with meaningful differences in interdental spacing in either arch.

**Table 3:** Interdental distances (mean ± SD) by feeding variable.

Feeding variable	Maxilla (mm)	Mandible (mm)	Observed trend
BF (any)	3.25 ± 2.15	2.38 ± 1.93	Slightly higher spacing in BF
BF >6 months	3.40 ± 2.17	2.40 ± 1.95	Trend toward greater spacing
FF (any)	3.23 ± 1.93	2.30 ± 1.90	No clear trend
BTL	3.40 ± 2.30	2.41 ± 1.72	Slight trend toward higher spacing
PAC	3.48 ± 2.42	2.58 ± 1.83	Slight trend toward higher spacing
CF	3.26 ± 1.70	2.15 ± 1.56	No clear trend

BF, breastfeeding; FF, formula feeding; BTL, bottle use; PAC, pacifier use; CF, complementary feeding. Values are mean ± SD. Trends describe direction; *p* > 0.05. Statistical tests: Mann–Whitney *U*, Student's *t*-test, Kruskal–Wallis.

**Figure 2:**
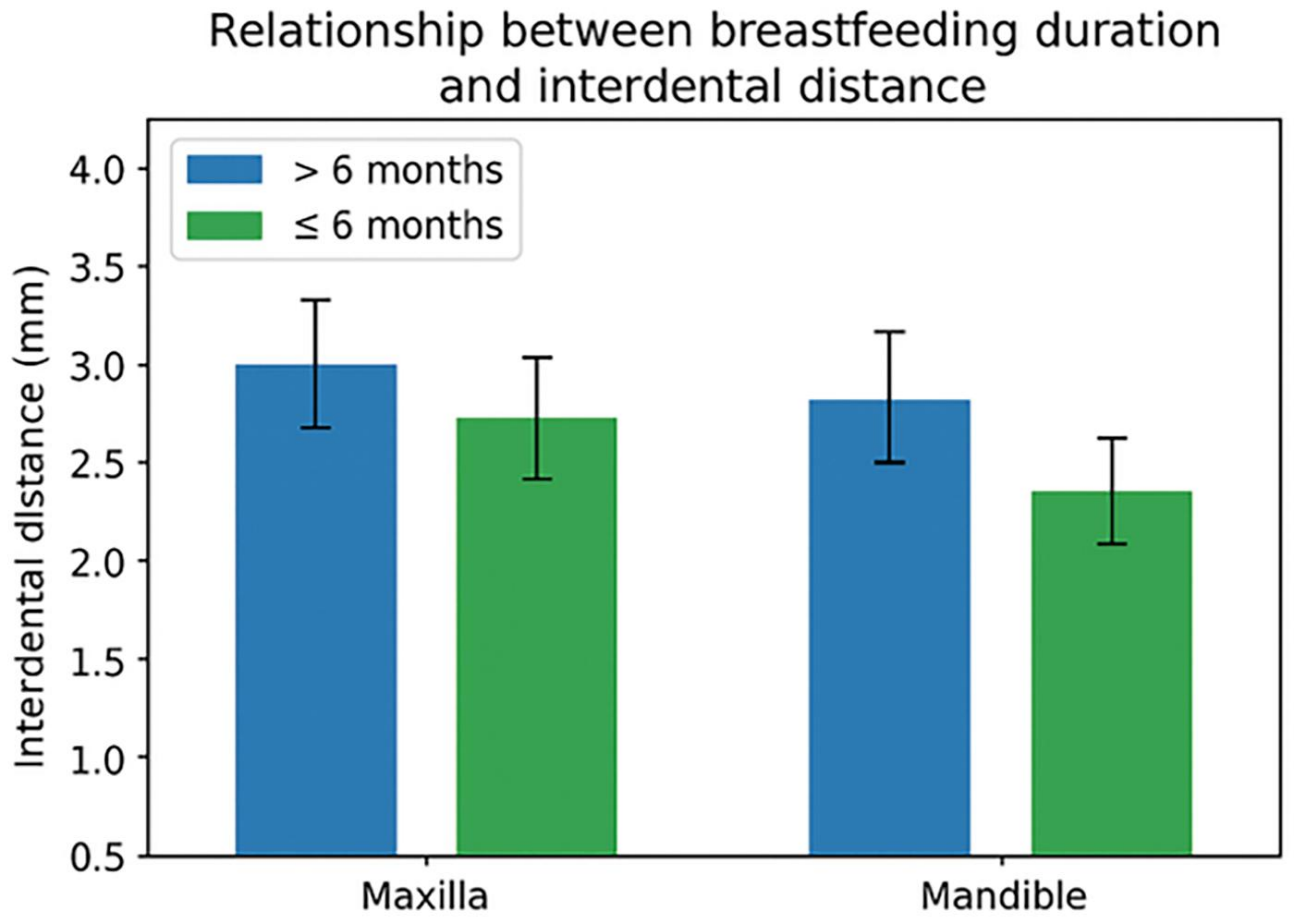
Mean interdental distance by breastfeeding duration (>6 vs. ≤6 months) ± SD. No significant differences (*p* > 0.05).

**Figure 3:**
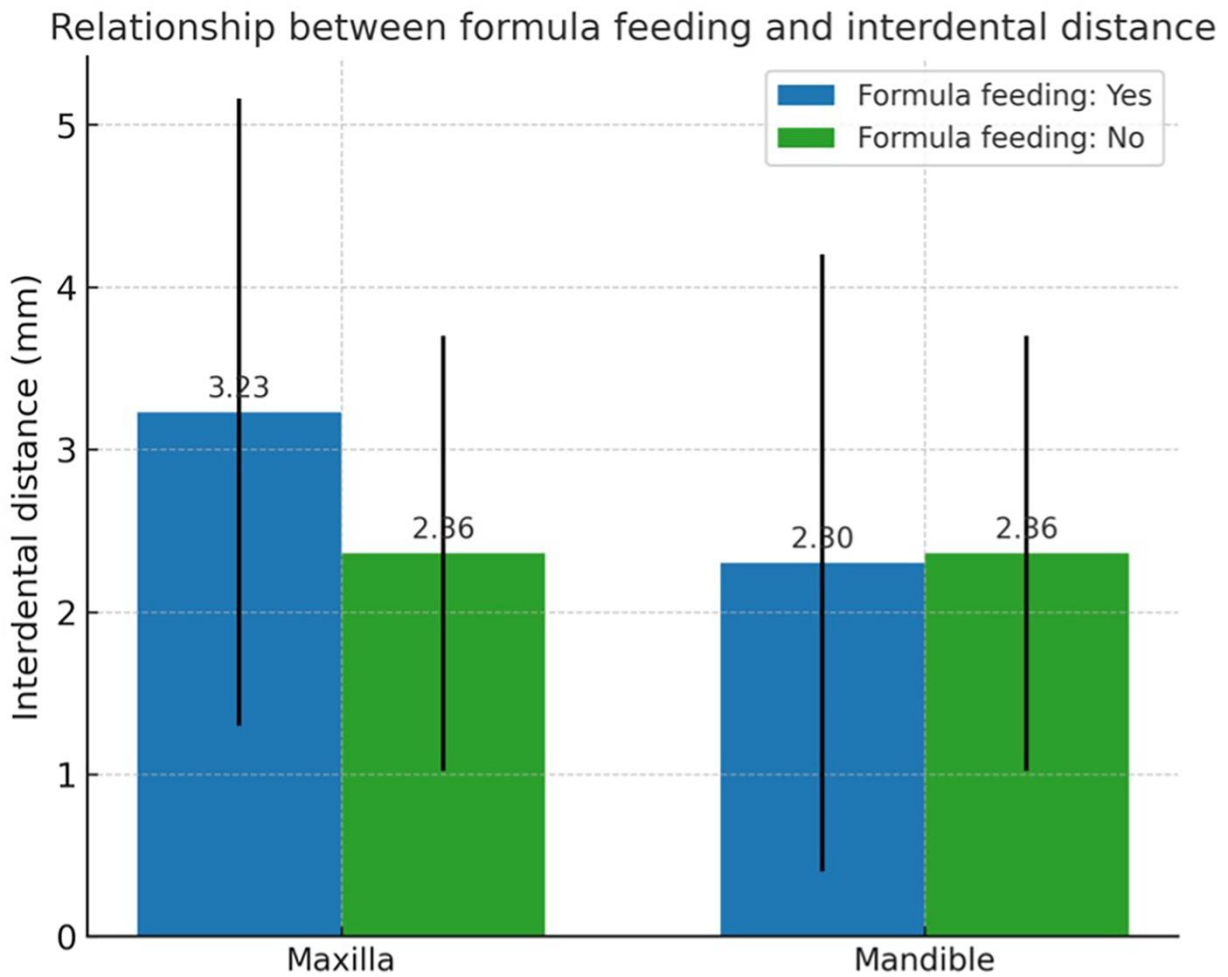
Mean interdental distance by formula feeding, bottle, and pacifier use ± SD. No significant differences (*p* > 0.05).

Figures 2, 3 illustrate these trends. Figure 2 shows interdental distances by breastfeeding duration (>6 vs. ≤6 months), and Figure 3 presents distances by formula feeding, bottle, and pacifier use. No statistically significant differences were observed in any comparisons (*p* > 0.05).

## Discussion

The present study analyzed the relationship between early feeding practices and interdental spacing in the primary dentition. Although no statistically significant associations were observed, a consistent trend toward larger interdental spaces in children with prolonged breastfeeding was noted. For example, mean maxillary interdental spacing in children breastfed >6 months was 3.40 ± 2.17 mm compared to 3.05 ± 1.82 mm in those breastfed ≤6 months, and mandibular spacing was 2.40 ± 1.95 vs. 2.20 ± 1.00 mm, respectively, suggesting a modest but consistent positive influence of breastfeeding on dental arch development (20). These results underscore the importance of early environmental factors in shaping dental arches, even when genetic factors remain dominant (1).

Physiologically, breastfeeding promotes greater orofacial muscle activity and proper tongue posture compared to bottle feeding, which may influence the shape and width of dental arches (4, 20). Masticatory forces generated during breastfeeding stimulate the development of jaw muscles, alveolar bone, and periodontal ligaments, contributing to optimal spacing (5). Diets requiring vigorous chewing, particularly hard or fibrous foods, further enhance jaw and muscle development, supporting adequate interdental spacing (21). Conversely, softer diets and prolonged bottle or pacifier use may reduce masticatory load, potentially leading to underdeveloped arches (16).

Normal interdental spacing is typically 0.5–2 mm, providing space for the eruption of permanent teeth (18, 22). In our cohort, mean interdental distances were within this physiological range, particularly in the primate areas of the maxilla (0.76 mm between the lateral incisor and canine) and mandible (0.36 mm between the canine and first molar) (19). Although differences between feeding groups were small and non-significant, they may still have practical implications; even modest variations in spacing could influence the likelihood of crowding during mixed dentition, especially in genetically predisposed individuals.

Our findings are generally consistent with previous studies highlighting the beneficial impact of prolonged breastfeeding (20, 23). Guerra et al. emphasized the role of extended breastfeeding in early craniofacial growth (20), and the Pan American Health Organization recommends breastfeeding for at least 6 months to prevent occlusal alterations (21). Some studies report statistically significant associations between breastfeeding duration and interdental spacing or related dental arch dimensions (25, 29). For instance, larger cross-sectional studies have shown that exclusive or prolonged breastfeeding (>6 months) is associated with significantly greater intercanine and intermolar widths in the primary dentition, which may indirectly reflect increased interdental spacing (24, 30, 31). Differences between these findings and the present results are likely explained by variations in sample size, population characteristics, and outcome definitions, as well as by the categorization of breastfeeding duration and feeding practices across studies. In particular, the relatively small sample size of the present study may have limited the ability to detect statistically significant differences despite observing similar directional trends.

Regarding oral habits, no significant effects of pacifier or bottle use were observed. This contrasts with reports showing that prolonged, intense non-nutritive sucking can reduce interdental spacing and promote anterior crowding (26, 27, 32, 33). In our cohort, the lack of association may be explained by relatively short duration or moderate frequency of dummy/bottle use, which may not have been sufficient to exert measurable effects on dental arch form.

A major strength of this study was the use of digital intraoral scanning and computer-assisted measurements, which provide high accuracy and reproducibility compared with traditional methods (32). The structured questionnaire allowed detailed assessment of early feeding practices and environmental influences.

Limitations include the small sample size (*n* = 52) and absence of *a priori* power calculation, increasing the risk of Type II error. The cross-sectional design precludes causal inference. Parental questionnaires introduce recall and perception bias (28, 34), and potential confounders, including socioeconomic status, parental education, and other oral habits, were not systematically controlled. Observed trends should therefore be interpreted cautiously.

Future research should focus on larger longitudinal studies, incorporating objective dietary assessment, standardized evaluation of complementary foods, and control for socioeconomic and educational factors. Longitudinal 3D imaging could track developmental changes in interdental spacing and jaw growth, clarifying subtle environmental effects and their clinical significance (21, 22).

## Data Availability

The raw data supporting the conclusions of this article will be made available by the authors, without undue reservation.
