# Supplementary figures and images for: Breastfeeding and interdental spacing in primary dentition: a digital cross-sectional study
Source: Front Dent Med. 2026 Mar 30;7:1716233. doi: 10.3389/fdmed.2026.1716233 (PMC13070942; doi:10.3389/fdmed.2026.1716233)

**Supplementary Material S1**


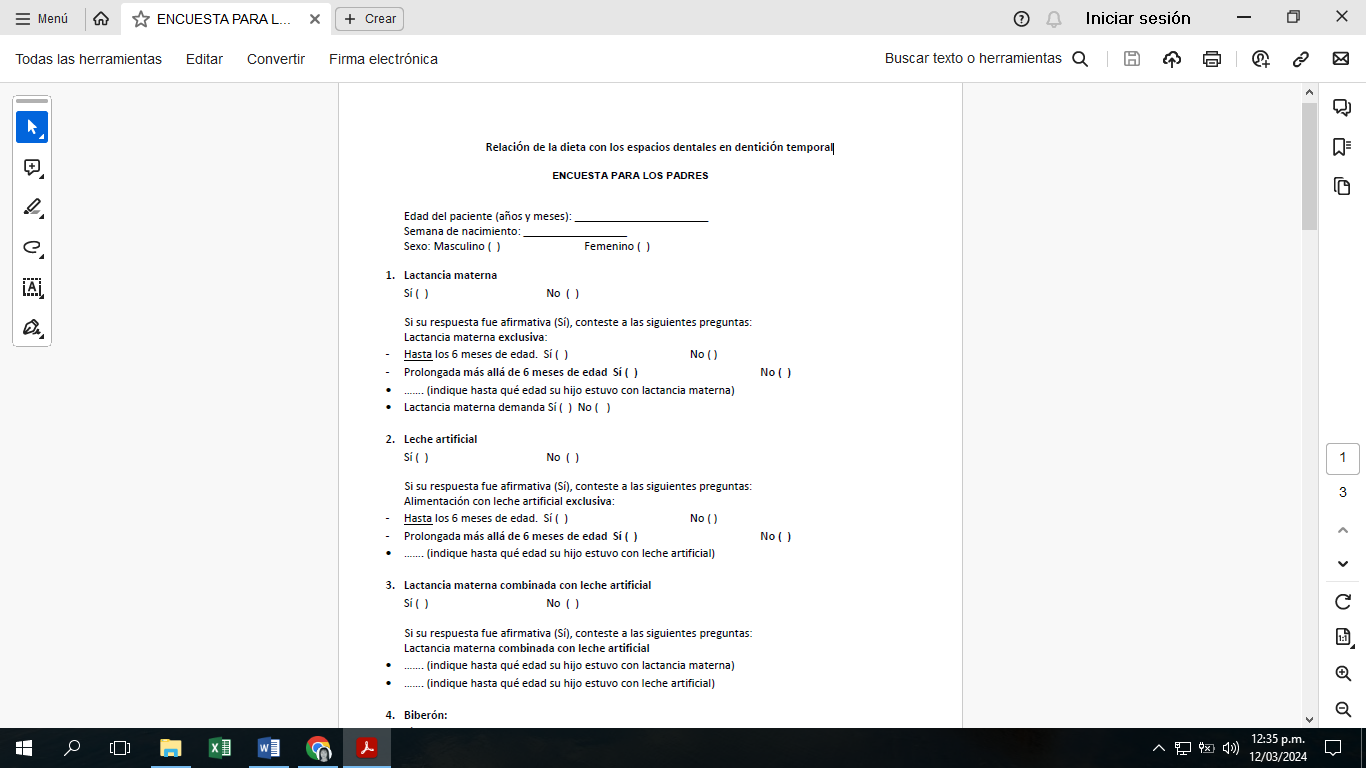


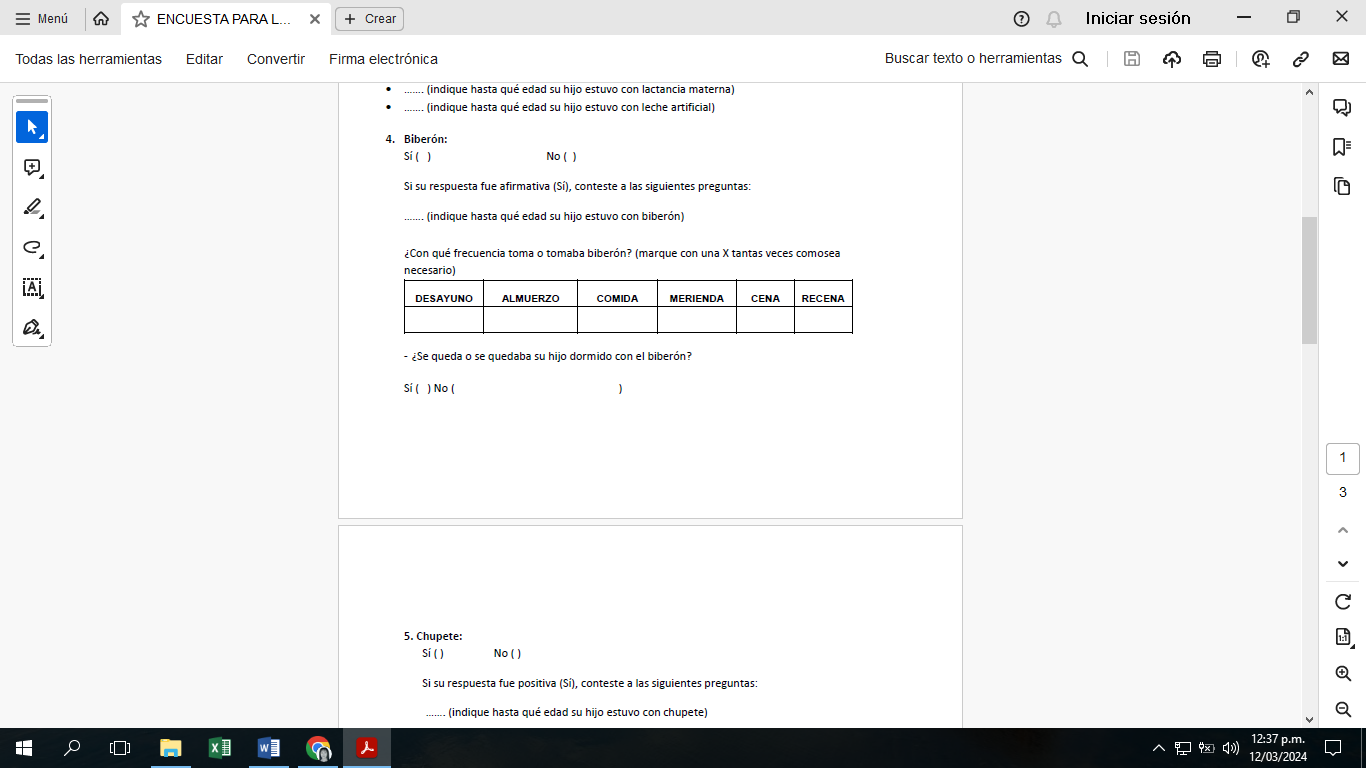


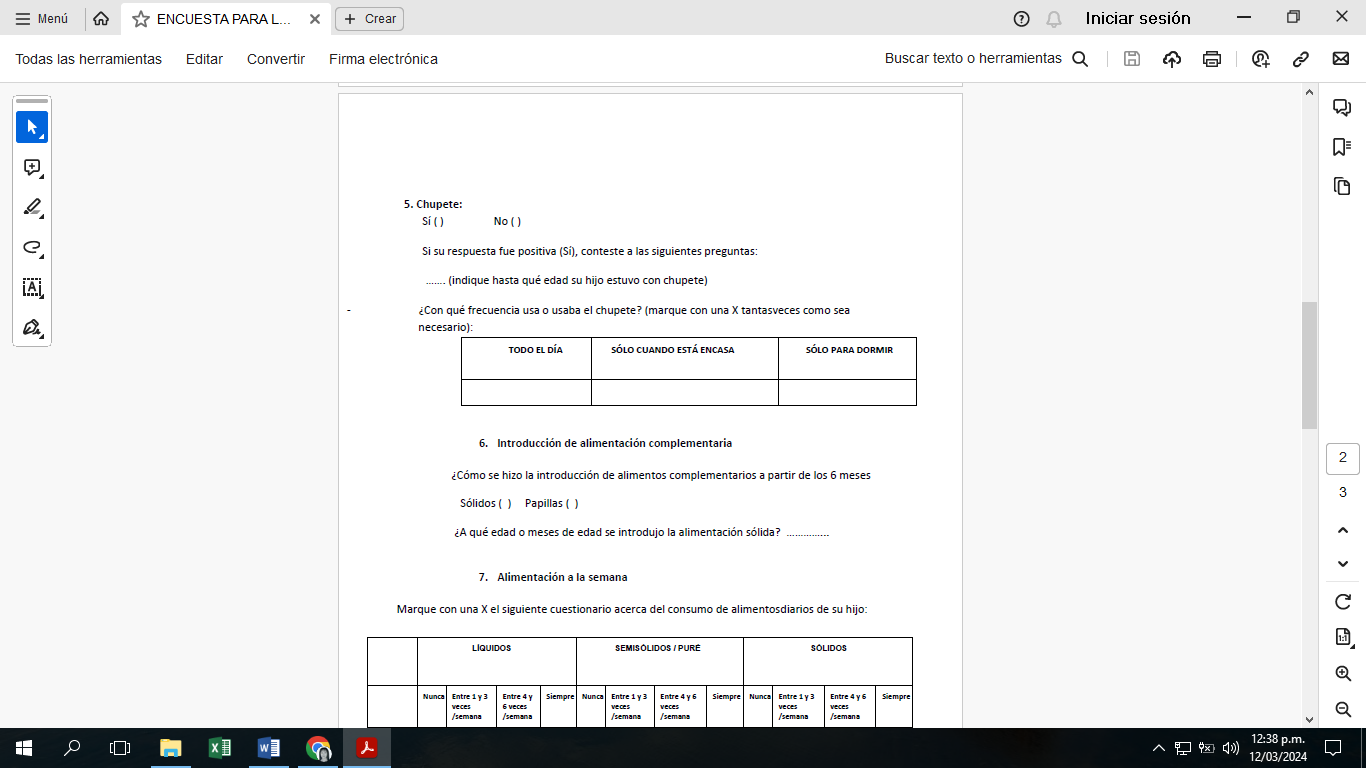


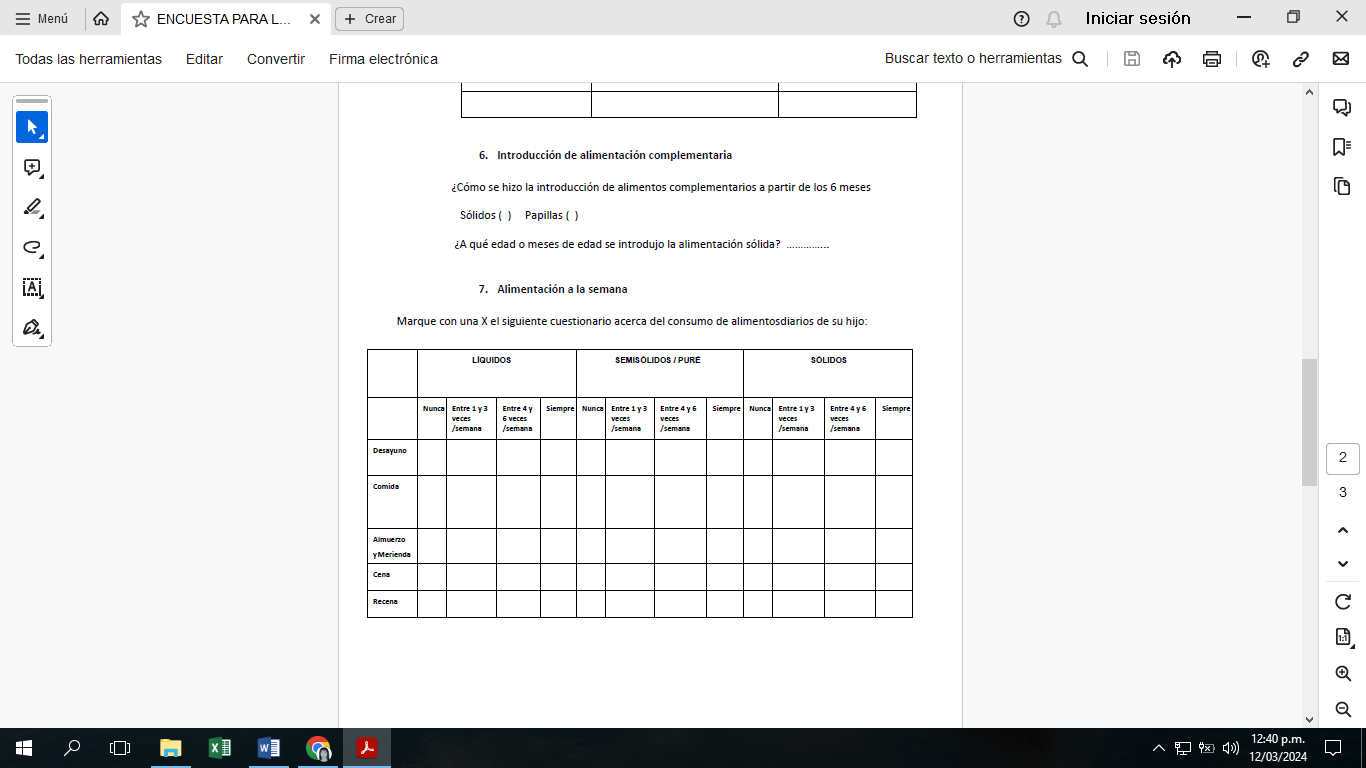

Supplement: Supplementary file 1 [file Supplementaryfile1.docx]
